# Evaluation of coal seam floor water bursting in multi-aquifer Gequan coal mine, China

**DOI:** 10.1038/s41598-022-23063-6

**Published:** 2022-10-27

**Authors:** Situ Lv, Yifan Zeng, Longqiang Zhang, Haonan Zhao

**Affiliations:** 1 National Engineering Research Center of Coal Mine Water Hazard Controlling, Beijing, 100083 China; 2 College of Geoscience and Surveying Engineering, China University of Mining and Technology (Beijing), Beijing, 100083 China; 3 China Energy Engineering Group Jiangsu Power Design Institute Co., Ltd., Nanjing, 211102 China

**Keywords:** Hydrology, Natural hazards, Engineering

## Abstract

A water-bursting evaluation of the coal seam floor is critical to ensure safety of coal mine production. The vulnerability index method based on AHP is selected for this study’s evaluation method. Water pressure, measured specific yield, equivalent thickness of effective aquiclude, brittle rock thickness under mining pressure damage zone, distribution of faults, distribution of collapse column, distribution of endpoints, and the intersection of fault are taken as the evaluation index based on the analysis of geological data in the study area. The authors assessed the threat posed by the two aquifers in the lower portion of the coal seam. Separate evaluations were conducted on the Benxi and Ordovician limestone aquifers. The results’ veracity was confirmed by comparing the obtained results to the water bursting point and a few boreholes. The evaluation results provide recommendations for the safe operation of coal mines.

## Introduction

Energy mix of a number of countries relies heavily on coal. One of these countries is China, which is abundant in the country’s resources. Nonetheless, the complex hydrogeological conditions in certain coalfields have exposed coal resources to the risk of water bursts at the coal mine’s floor during the mining process^1–3^. Consider the Hanxing mining area in northern China as an example. Several thin limestone aquifers and a highly water-rich Ordovician limestone aquifer are present in the lower portion of the Carboniferous Permian coal seam, where mining is currently taking place. In the Hanxing mining area, complex hydrogeological conditions have caused several large-scale water bursting incidents on the mine floor. These water bursting incidents endanger the lives of coal miners and cause substantial harm to coal producers^4^.

Before mining, it is essential to evaluate the coal seam floor for water bursting. The sudden appearance of water at the bottom of a coal seam is due to a combination of factors. After numerous experiences with water damage in the bottom slab, some European countries realised at the turn of the twentieth century that the problem was mitigated when there was a specific lithological stratum in the bottom slab of the coal seam and that the thicker the “stratum” was, the less the impact of the water breakout; they termed this a “water barrier”. Between 1940 and 1949, the Hungarian scholar W. France discovered a relationship between the bottom slab and the pressure of the aquifer. He coined the term “relative water barrier” to describe the ratio of the thickness of the water barrier to the head pressure. When the ratio is less than 1.5 m/atm, a sudden water flow may occur in the bottom slab. Other countries have adopted 2 m/atm as the threshold value for judging a sudden water flow in the bottom slab, and it is widely used. In the same period, B. Slesarev discovered that when the bottom aquifer had a certain head height, the bottom slab would burst into the water and derived the theoretical formula for safe head height via the “super-stationary beam model” confirmed in the subsequent water control work^5–8^. Chinese scholars, inspired by foreign scholars, have proposed various evaluation methods, with the sudden water coefficient method and the vulnerability index (VI) method being the most popular^9–14^. By calculating the water pressure per unit thickness of the aquifer at the base of a coal seam, the water inrush coefficient method is used to determine whether there is an emerging risk in the calculation area. This method has the advantage of being simple and easy to use, and the evaluation results can be obtained quickly; however, it considers too few factors, resulting in inaccurate evaluations in some regions. The VI method analyses the factors influencing coal mines’ water hazard. After calculating the weights of the influencing factors using the weight calculation method, the influencing factors are mapped using geographic information system (GIS). The calculated area’s VI is then obtained. Because the VI method can account for a broader range of influencing factors, the final evaluation result will be more precise^15,16^. Both methods contribute significantly to the safety of coal mines.

To evaluate the threat of water bursting at the coal seam floor during production in the Gequan coal mine, the authors selected the vulnerability index method as the evaluation method for this study. Analysis of the hydrogeological data and water bursting point in the study area revealed that both the Benxi limestone aquifer and the Ordovician limestone aquifer threaten the 9# coal seam. The authors selected water pressure, measured specific yield, equivalent thickness of effective aquiclude, brittle rock thickness under mining pressure damage zone, distribution of faults, distribution of collapse column, distribution of endpoints, and the intersection of the fault and fault-scale index as the evaluation of the impact factors to obtain accurate evaluation results. Both Benxi limestone and Ordovician limestone were subjected to separate evaluations. The accuracy of the evaluation results was demonstrated by comparing the obtained results to the known water bursting point and a few boreholes.

## Study area

### Location

**Figure 1:**
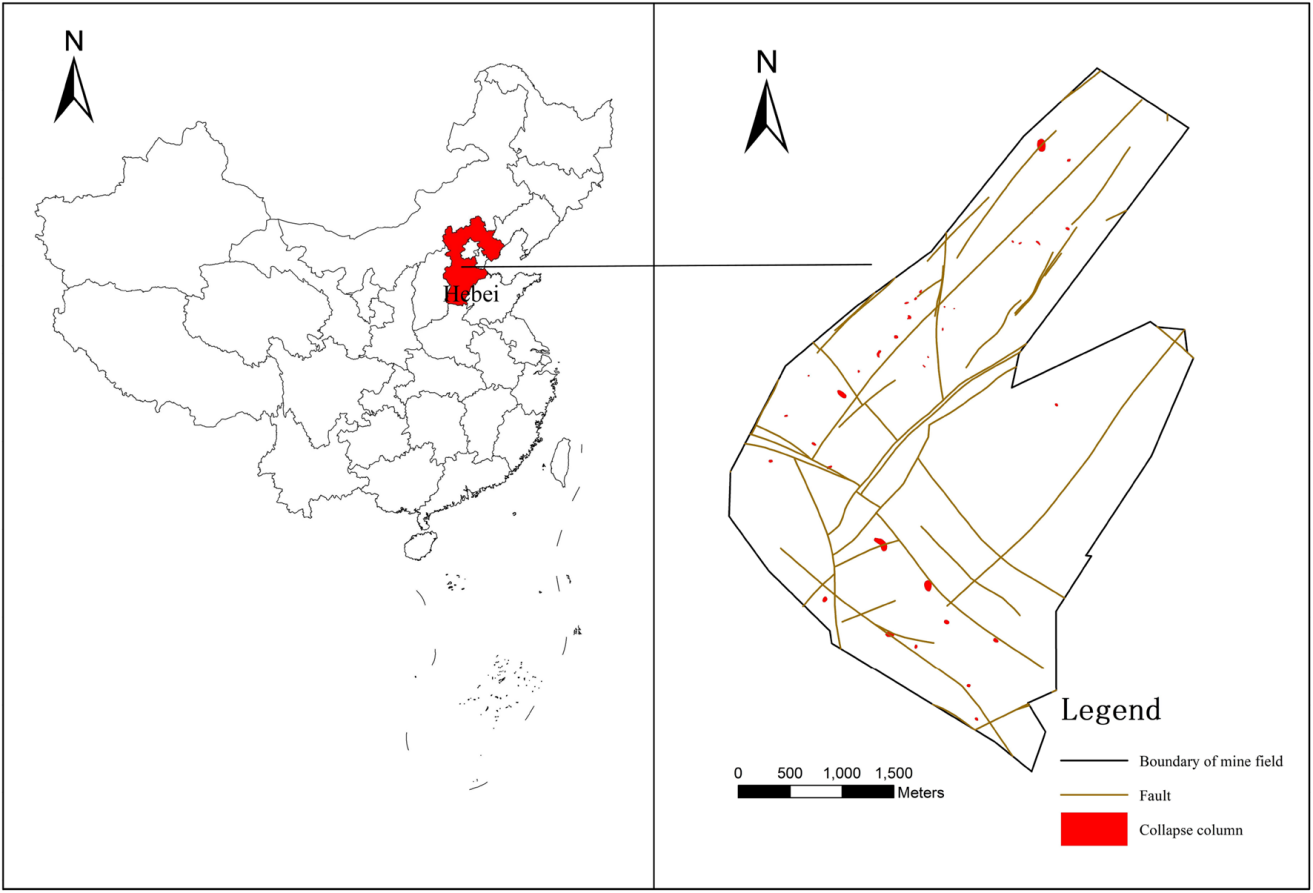
Location and structural geology map, Images are created using the Coreldraw, http://www.coreldraw.com/en/?link=wm.

Gequan coal mine is located approximately 18 kms south-southwest of Xingtai City, Hebei Province. Its administrative division falls under the jurisdiction of Shili Ting Town, Shahe City, and Xingtai City (Fig. 1). The central geographical coordinates are 114°20′50″ ~ 114°23′52″ East and 36°55′20″ ~ 36°59′00″ North. The mine is situated in the pre-hill region of the Taihang Mountains, where gullies and valleys are forming. The terrain is elevated in the south and low in the north, with a ground elevation of + 92 ~  + 190 m. Gequan Mine is situated in a semiarid, warm-temperate, continental monsoon climate zone with four distinct seasons, with dry winters and wet summers. Most atmospheric precipitation occurs between July and September, with a multi-year average temperature of approximately 13 °C. The hottest and coldest months of the year are July and late December to early January, respectively. The freezing season lasts from November to February, with a maximum depth of 0.44 m. The average annual wind direction is north-westerly, with a maximum annual wind speed of 18 m/s.

### Geology and hydrogeology

The surface of the Gequan mine is composed of Cenozoic Quaternary sedimentary layers in angular unconformable contact with the underlying rock formations. From oldest to youngest, the strata developed in the mining field are as follows: Majiagou Formation and Fengfeng Formation of the middle Ordovician system; the middle Benxi Formation and upper Taiyuan Formation of the Carboniferous system; the Lower Shanxi Formation, Lower Shihezi Formation, and upper Shihezi Formation of the Permian system; and the Quaternary system.

The study area contains the Quaternary pore aquifer, the Permian sandstone fissure aquifer, the Carboniferous thin limestone fissure karst aquifer, and the Ordovician limestone fissure karst aquifer.

### Analysis of water bursting in the 9# coal seam

**Figure 2:**
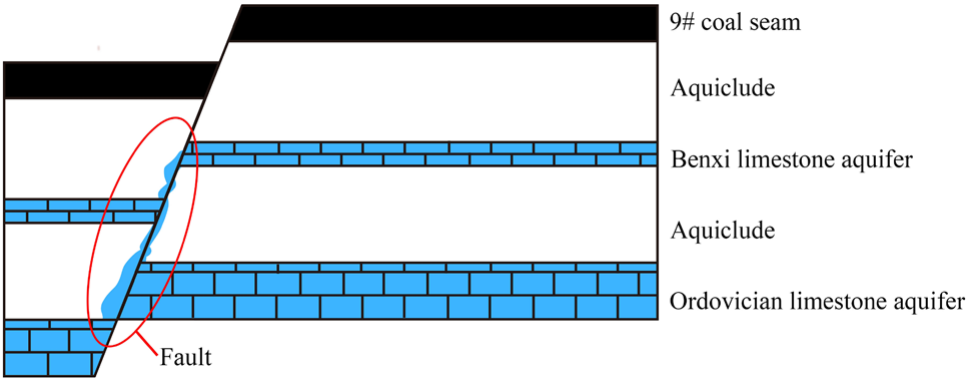
The relative position of the strata.

This study examines the 9# coal seam and its lower aquifer. All 9# coal seams in the study area can be mined using the longwall mining method with sublevel caving, with the thinnest point measuring 0.83 m and the thickest measuring 8.7 m. The Benxi limestone aquifer and the Ordovician limestone aquifer exist beneath the 9# coal seam on average, and the Benxi limestone aquifer is only 9# coal seam on average, with high water-richness in the local area. The aquiclude between the Benxi Limestone aquifer and the Ordovician Limestone aquifer is between 8.08 m and 16.68 m, with an average of 13 m, and is easily connected hydraulically. The fault is the primary conduit through which the Ordovician Limestone aquifer recharges the Benxi Limestone aquifer, according to hydrogeological tests (Fig. 2). There were two large scale water surges from the Ordovician limestone aquifers in the study area. One was during the mining of the #2 coal seam, when a hydraulic connection with the Ordovician limestone aquifers was discovered; the other was when the mine pressure damage zone generated by the mining of the #9 coal seam came into contact with the Ordovician limestone aquifers. The water surges occurred beneath the hydraulic support at the 1293 working face. The initial surge was only 5 m^3^/h, and no control measures were implemented, but one day later, the surge increased to 288 m^3^/h. The surge was later maintained at 130 m^3^/h after slurry control, and it was later determined through water quality testing that the source of the water surge was the Ordovician limestone aquifers. It was the only water surge incident during the mining of the 9# coal seam. In conclusion, the Ordovician limestone aquifer and the Benxi limestone aquifer will be the subject of this coal seam floor evaluation, and fault and mine pressure damage zones have become a vital focus.

## Results

### Weight calculation results

**Figure 3:**
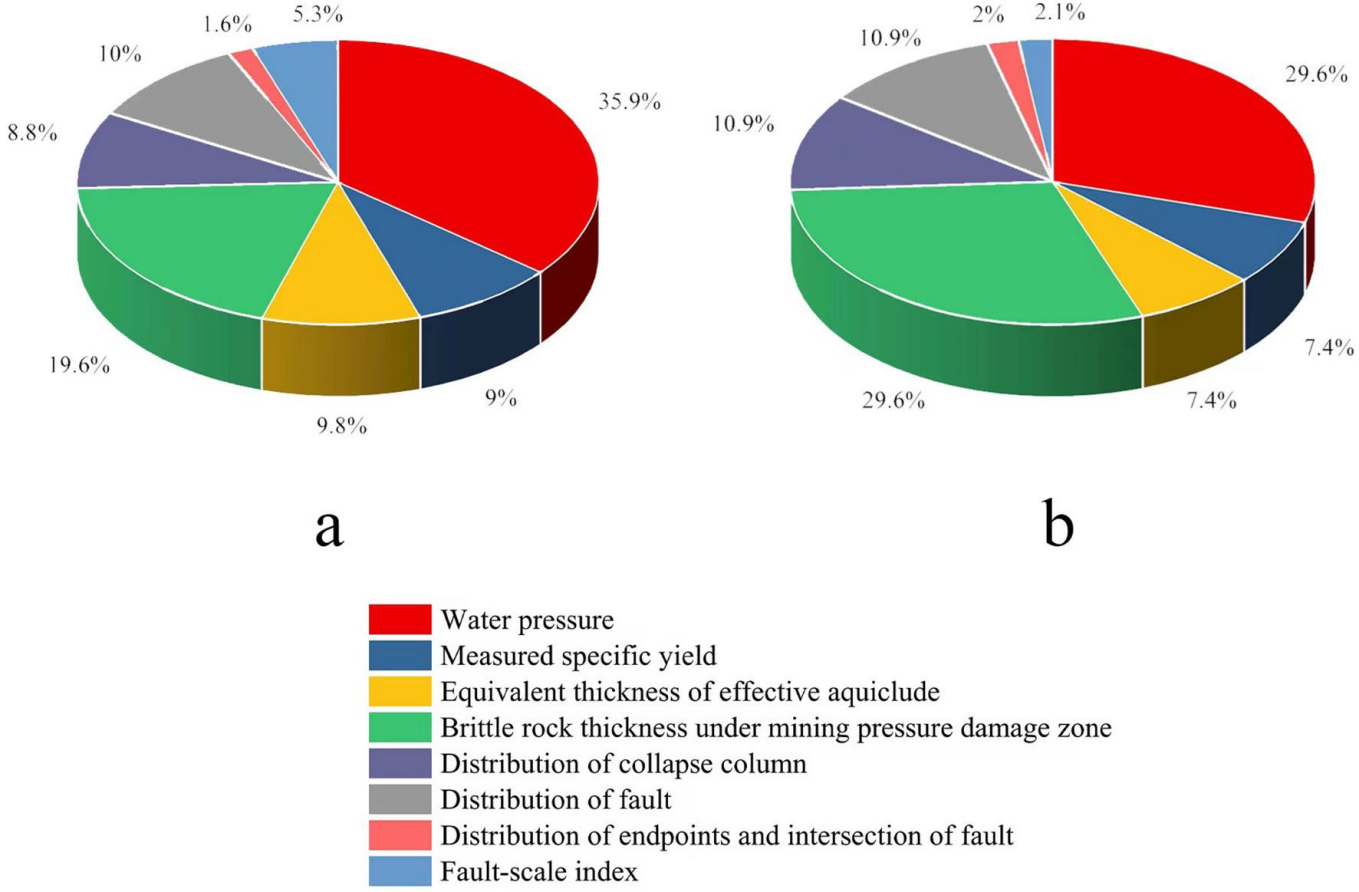
Pie chart of influence factor weights: (**a**) Ordovician limestone aquifer, (**b**) Benxi limestone aquifer.

Figure 3 depicts the weight of various influencing factors determined by the AHP weight method.

### Results of the water bursting evaluation

The authors then constructed the vulnerability index equation for 9# coal seam after obtaining the weights of each influencing factor. The Ordovician limestone aquifer is represented by Eq. (1), while the Benxi limestone aquifer is represented by Eq. (2).

1 $$\mathrm{VI}=\sum_{\mathrm{i}=1}^{\mathrm{m}}{\mathrm{W}}_{\mathrm{i}}{\mathrm{f}}_{\mathrm{i}}\left(\mathrm{x},\mathrm{y}\right)=0.3591\cdot {\mathrm{f}}_{1}\left(\mathrm{x},\mathrm{y}\right)+0.0898\cdot {\mathrm{f}}_{2}\left(\mathrm{x},\mathrm{y}\right)+0.0980\cdot {\mathrm{f}}_{3}\left(\mathrm{x},\mathrm{y}\right)+0.1961\cdot {\mathrm{f}}_{4}\left(\mathrm{x},\mathrm{y}\right)+0.0877\cdot {\mathrm{f}}_{5}\left(\mathrm{x},\mathrm{y}\right)+0.1004\cdot {\mathrm{f}}_{6}\left(\mathrm{x},\mathrm{y}\right)+0.0157\cdot {\mathrm{f}}_{7}\left(\mathrm{x},\mathrm{y}\right)+0.0532\cdot {\mathrm{f}}_{8}\left(\mathrm{x},\mathrm{y}\right)$$

2 $$\mathrm{VI}=\sum_{\mathrm{i}=1}^{\mathrm{m}}{\mathrm{W}}_{\mathrm{i}}{\mathrm{f}}_{\mathrm{i}}\left(\mathrm{x},\mathrm{y}\right)=0.2963\cdot {\mathrm{f}}_{1}\left(\mathrm{x},\mathrm{y}\right)+0.0741\cdot {\mathrm{f}}_{2}\left(\mathrm{x},\mathrm{y}\right)+0.0741\cdot {\mathrm{f}}_{3}\left(\mathrm{x},\mathrm{y}\right)+0.2963\cdot {\mathrm{f}}_{4}\left(\mathrm{x},\mathrm{y}\right)+0.1095\cdot {\mathrm{f}}_{5}\left(\mathrm{x},\mathrm{y}\right)+0.1095\cdot {\mathrm{f}}_{6}\left(\mathrm{x},\mathrm{y}\right)+0.0196\cdot {\mathrm{f}}_{7}\left(\mathrm{x},\mathrm{y}\right)+0.0207\cdot {\mathrm{f}}_{8}\left(\mathrm{x},\mathrm{y}\right)$$

**Figure 4:**
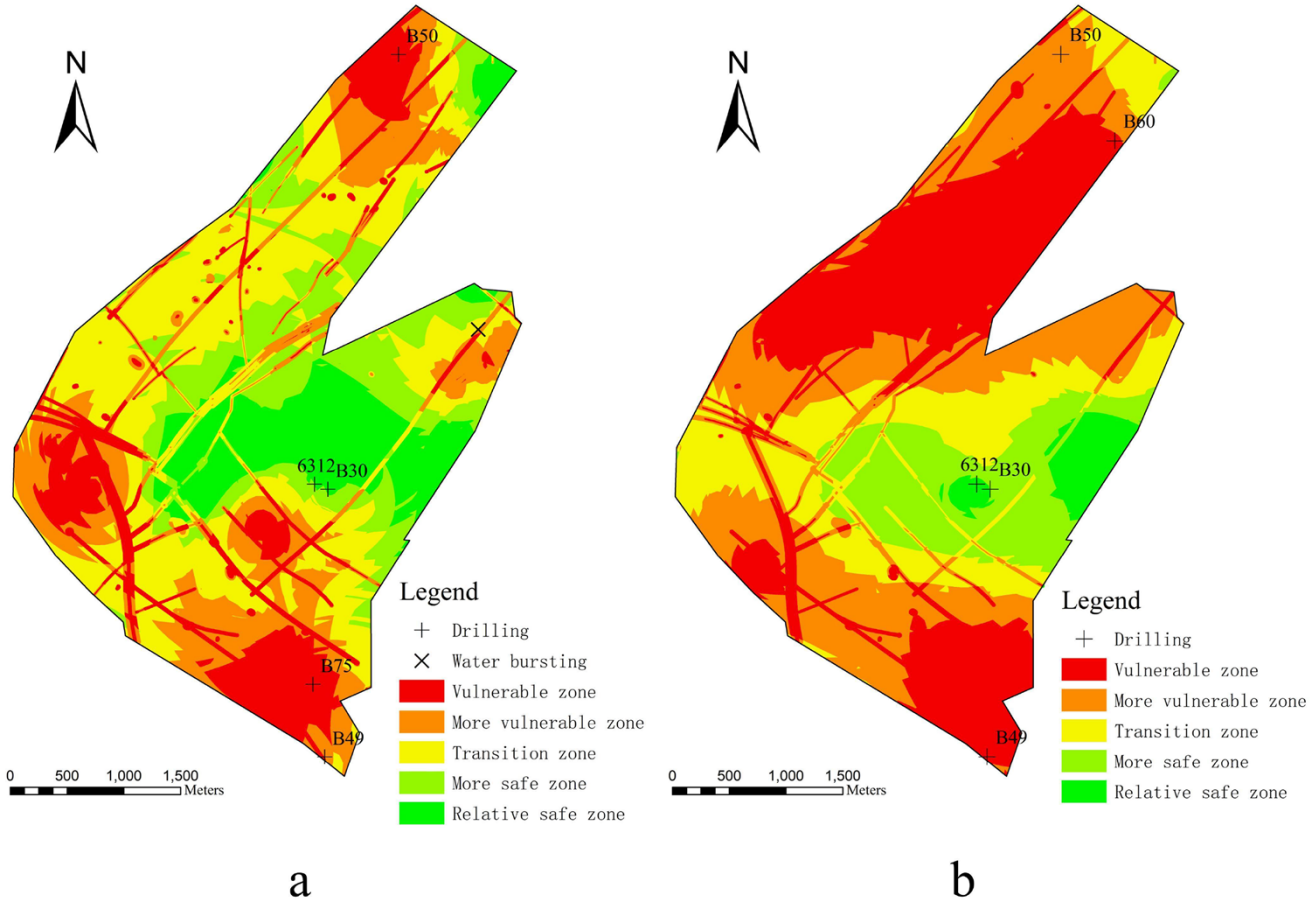
Map of water bursting evaluation results: (**a**) Ordovician limestone aquifer; (**b**) Benxi limestone aquifer. Images are created using the ArcGis, https://www.esri.com/en-us/arcgis/products/index.

Then, Natural Jenks, the default classification method in ArcGIS, was utilised to categorise all WI index data into five grades: vulnerable, more vulnerable, transition, more safe, and relative safe. The water burst hazard mapping of the 9# coal seam in the study area was created using GIS (Fig. 4). The evaluation results graph indicates the risk of water bursting at the floor of the 9# coal seam in the Gequan coal mine is high. Due to its proximity to the coal seam, the Benxi aquifer has a much larger susceptible zone. The southern portion of the study area is more at risk from the Benxi and Ordovician aquifers. The Benxi aquifer is threatened more in the central portion of the study area, while the Ordovician aquifer is threatened more at the northern boundary of the study area. Most faults and collapse columns are in the vulnerable zone, which is consistent with the results of the previous analysis of water bursting.

## Discussion

By analysing the geological data of the Gequan coal mine, we proposed using the vulnerability index method to evaluate water bursting in 9# coal seams and the Ordovician limestone aquifer and Benxi limestone aquifer, respectively. It is because the coal mine faces the threat of water damage from multiple aquifers during production. The study area was divided into five classes using the natural classification method. To verify the accuracy of the evaluation results, we plotted the known point of burst water in the target coal seam and a few boreholes with distinguishing characteristics on the map and evaluated the model’s accuracy based on whether these points were in the optimal grade area. There is currently only one burst water point within the study area, so the remaining validation points have been replaced with boreholes. The resulting map reveals the water intrusion point within the vulnerable zone. The thickness of brittle rock beneath the mine pressure damage zone at B50 and B75 is approximately 1 m, whereas the thickness of brittle rock beneath the mine pressure damage zone at B49 is approximately 3.3 m, and the water pressure is greater. The said drill holes are in the vulnerable zone, proving that the evaluation results are more precise^17^.

**Figure 5:**
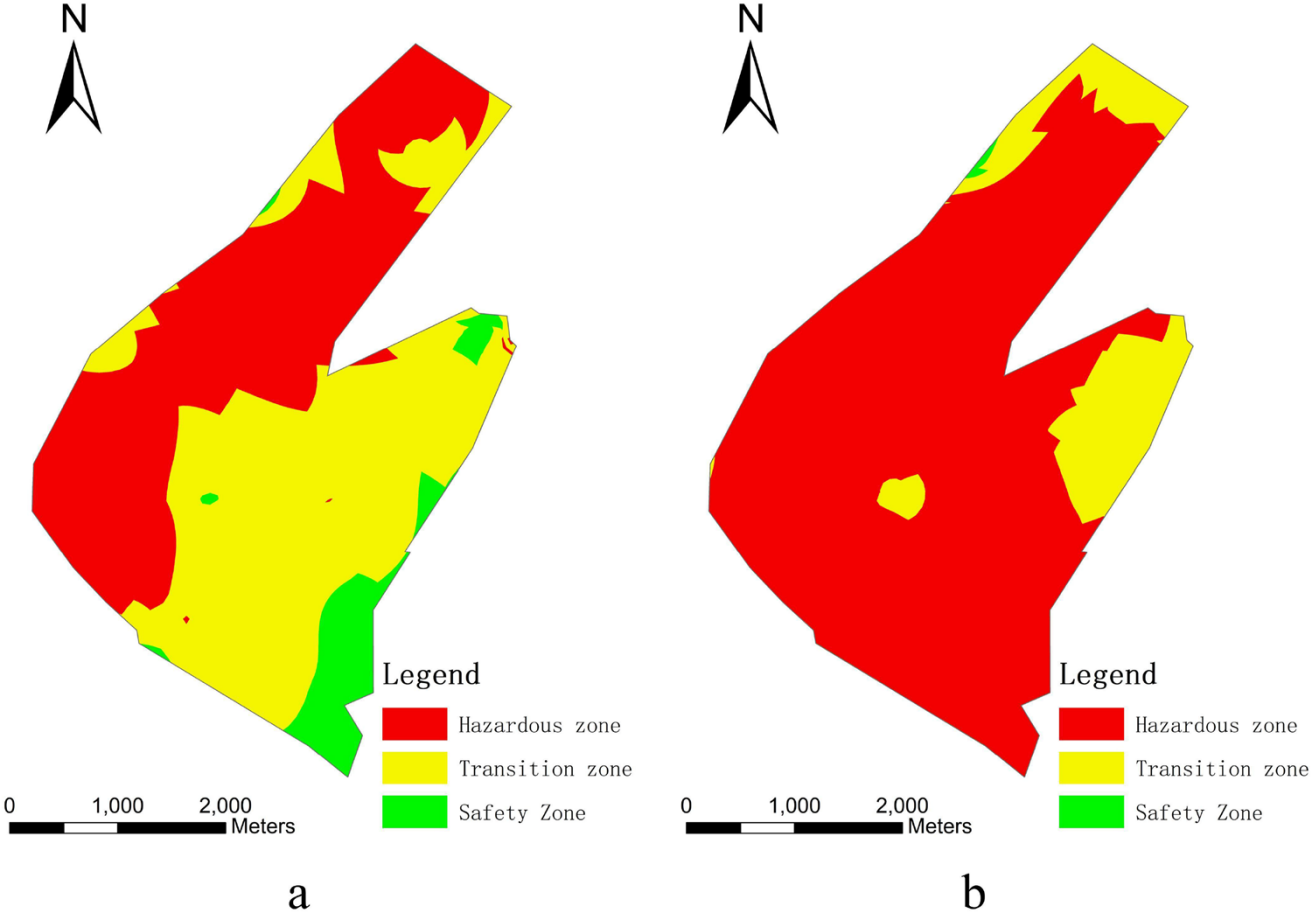
Map of water inrush coefficient method results: (**a**) Ordovician limestone aquifer; (**b**) Benxi limestone aquifer. Images are created using the ArcGis, https://www.esri.com/en-us/arcgis/products/index.

In terms of evaluation methods, most coal producers currently employ the water inrush coefficient to determine the mining safety in the target area. However, the water inrush coefficient only considers the water barrier and water pressure. It also does not consider important information such as mine pressure damage zone, water-richness, and geological structure. Hence, the obtained evaluation results are not comprehensive and are only suitable for simple site condition evaluation. Comparing the results of the two methods reveals that their zoning is comparable (Fig. 5). However, the vulnerability index method’s results are more detailed and exhaustive because it considers more factors and has more classes. Therefore, the classification of certain regions varies. For instance, the northern portion of the study area is classified as vulnerable by the VI method but safe by the water inrush coefficient method. It is because the VI method accounts for the zone of mine pressure damage on the thickness of the water barrier, while the water inrush coefficient method does not. In addition, because the vulnerability index method takes geological structure into account, the planform of the geological structure is visible on the results map.

At this stage, AHP is one of the most popular weight calculation methods for evaluating water bursting. The primary basis for obtaining weights using this method is the production workers’ experience in the study area. Although this method of calculating weight takes more into account the subjective ideas of individuals, field experience is essential for accidents with more complex causes, such as water bursting in coal mines. Currently, there are numerous exhaustive weight calculation methods, such as combining AHP and objective weight calculation methods, multiplying the weight values obtained by the two methods by different coefficients, and adding the weight values obtained by the two methods to obtain the final weight value^18^. It can prevent the weight results from being too subjective, but it has certain drawbacks. Most objective weighting methods are based on the degree of data variation. The more significant the data fluctuation, the greater the weighting value; however, this has significant drawbacks in practice. For instance, if the water pressure in the aquifer is exceptionally high but varies very little across the entire study area, the weighting of water pressure’s influence would be small, but this is not realistic.

In summary, for evaluating coal seam floor water bursting, the vulnerability index method yields more comprehensive results than the water inrush coefficient method. The geological structures, which are a significant cause of water damage accidents, are not considered by the sudden water coefficient method, which significantly reduces the precision of the results. Due to the lack of target coal seam outbursts within the study area at this time, it is questionable whether a single outburst can prove the model’s accuracy; however, the inclusion of characteristic boreholes provides some support for the model’s accuracy. As the coal seam continues to be extracted in the final stages, the emergence of additional water flare points will significantly assist in validating and revising the evaluation model. As one of the most prevalent weighting methods at this stage, AHP considers the engineers’ experience in the study area, bringing the results closer to production reality and preventing inaccurate evaluation results caused by excessive focus on numerical changes in the data. This study’s results have implications for future production plans in the study area.

## Conclusions

By analysing the hydrogeological data of Gequan Mine, this paper concludes that the Benxi limestone aquifer and the Ordovician limestone aquifer in the lower portion of the 9# coal seam pose threats to mining operations and that the Ordovician aquifer recharges the Benxi aquifer via faults. To obtain precise evaluation results, the water inrush coefficient method, which only considers the thickness of the water barrier and the water pressure, is no longer suitable for this study; therefore, the vulnerability index method is used. By natural discontinuity classification, the VI area was divided into five grades: vulnerable, more vulnerable, transitional, more safe, and relatively safe. Through the evaluation of the burst water on the floor of the study area, we have gained an understanding of the water damage problems that may be encountered during mining, which will be helpful for future mining at Gequan Mine and evaluation work in other coal mines.

## Methodology

After compiling and analysing the geological and hydrogeological data of the study area, this study selects the following influencing factors for evaluation: water pressure, measured specific yield, equivalent thickness of effective aquiclude, brittle rock thickness under mining pressure damage zone, distribution of faults, distribution of collapse column, distribution of endpoints, and the intersection of the fault and fault-scale index. Due to the uneven distribution of strata, different aquifers have different effects on the same coal seam; therefore, this evaluation was conducted separately for the Ordovician and Benxi aquifers. The AHP-based vulnerability index method was selected as the evaluation method to obtain accurate evaluation results. Since only one water outburst from the Ordovician aquifer occurred in the 9# coal seam, a portion of the geological borehole was used as the validation point.

### Influencing factors for water bursting

#### Water pressure

The water pressure in an aquifer is one of the most influential factors in the bursting of water at the coal seam floor. It is because the water level elevation of the aquifer must be higher than the elevation of the coal seam floor for water to burst through the coal seam floor.

#### Measured specific yield

Specific yield can respond to the water-richness of an aquifer, but only if the aquifer is highly water-rich can water bursting occur. In order to unify the standard, this study selected the equivalent thickness of effective aquiclude when the borehole hole diameter is 91 mm, and the water level drops 10 m^19^.

#### Equivalent thickness of effective aquiclude

The aquiclude at the base of the coal seam acts as a water bursting suppressor, and its water barrier capacity is proportional to the barrier’s thickness and rock type. As determined by field experiments, effective aquiclude thickness equals total aquiclude thickness minus the thickness of the mining pressure damage zone. As the aquiclude is composed of various rocks, the water-blocking capacity of these rocks varies. To evaluate the water barrier capacity of various rocks, Chinese researchers have compiled their engineering knowledge and proposed a conversion factor for rock thickness, these data are presented in Table 1. In this case, the equivalent thickness is equal to the sum of the thickness of each rock layer multiplied by the conversion factor^20^. The thickness of the effective water aquiclude is calculated using Eq. (3). The equivalent thickness is calculated using Eq. (4).

**Table 1:** Conversion factor for the thickness of rock.

Lithology	Sandstone	Limestone	Sandy shale	Mudstone	Crushed zone
Comprehensive equivalent coefficient	1.1	1.2	0.7	0.6	0.3

3 $${\mathrm{h}}_{2} =\mathrm{ H}-{\mathrm{h}}_{1}$$

4 $${\mathrm{h}}_{2}^{,} = \sum_{\mathrm{i}=0}^{\mathrm{n}}{\upmu }_{\mathrm{i}}{\mathrm{h}}_{\mathrm{i}2}$$

${h}_{2}$ is the thickness of effective aquiclude; H is the thickness of aquiclude; ${\mathrm{h}}_{1}$ is the thickness of mining pressure damage zone; is the equivalent thickness of effective aquiclude; is the conversion factor for the rock thickness; and is the rock thickness.

#### Brittle rock thickness under mining pressure damage zone

The location of aquiclude’s rock formations affects water bursting. In the presence of brittle rocks such as sandstone and limestone, an aquiclude is more resistant to pressure due to its hard rock characteristics. The location of brittle rocks affects their resistance to water and pressure differently. Only when the brittle rock is situated in a productive aquifer does it play a significant role in water resistance. When brittle rock is located within the mine pressure damage zone after a coal seam has been mined, mine pressure damage causes fractures in the brittle rock but does not affect water resistance.

#### Distribution of fault

Fault disrupts the integrity of aquiclude at the coal seam floor. During fault formation, many fissures are formed through which water pressure will direct groundwater upwards^21,22^. During the development process, the fault forms not only a fracture zone but also an affected zone surrounding the fracture zone. The fault fracture zone is the area between two fault discs where the rock layer is severely misplaced, loses its structural integrity, and cannot effectively isolate water when it is not filled. The fault-affected zone is located on both sides of the fracture zone and is characterised by the development of fractures and high water conductivity. Therefore, it is necessary to calculate the width of the fracture zone and the affected zone when quantifying faults. The width of the fracture zone is determined by geological data, whereas the width of the affected zone must be calculated using empirical formulas (Eq. 5).

5 $${\mathrm{k}}_{\mathrm{y}}=\upgamma {\mathrm{h}}^{3/5}$$

k_y_ is the normal fault affected zone width; γ is the coefficient related to the lithology of both plates in the normal fault. When the discs are composed of soft rock and coal or other loose rock layers, the correlation coefficient is 1.14. The correlation coefficient is 0.76 when all normal faults are composed of layers of medium-hardness rock. When two layers of hard rock separate normal faults, the coefficient is 0.38. Finally, h is the normal fault drop^23^.

**Figure 6:**
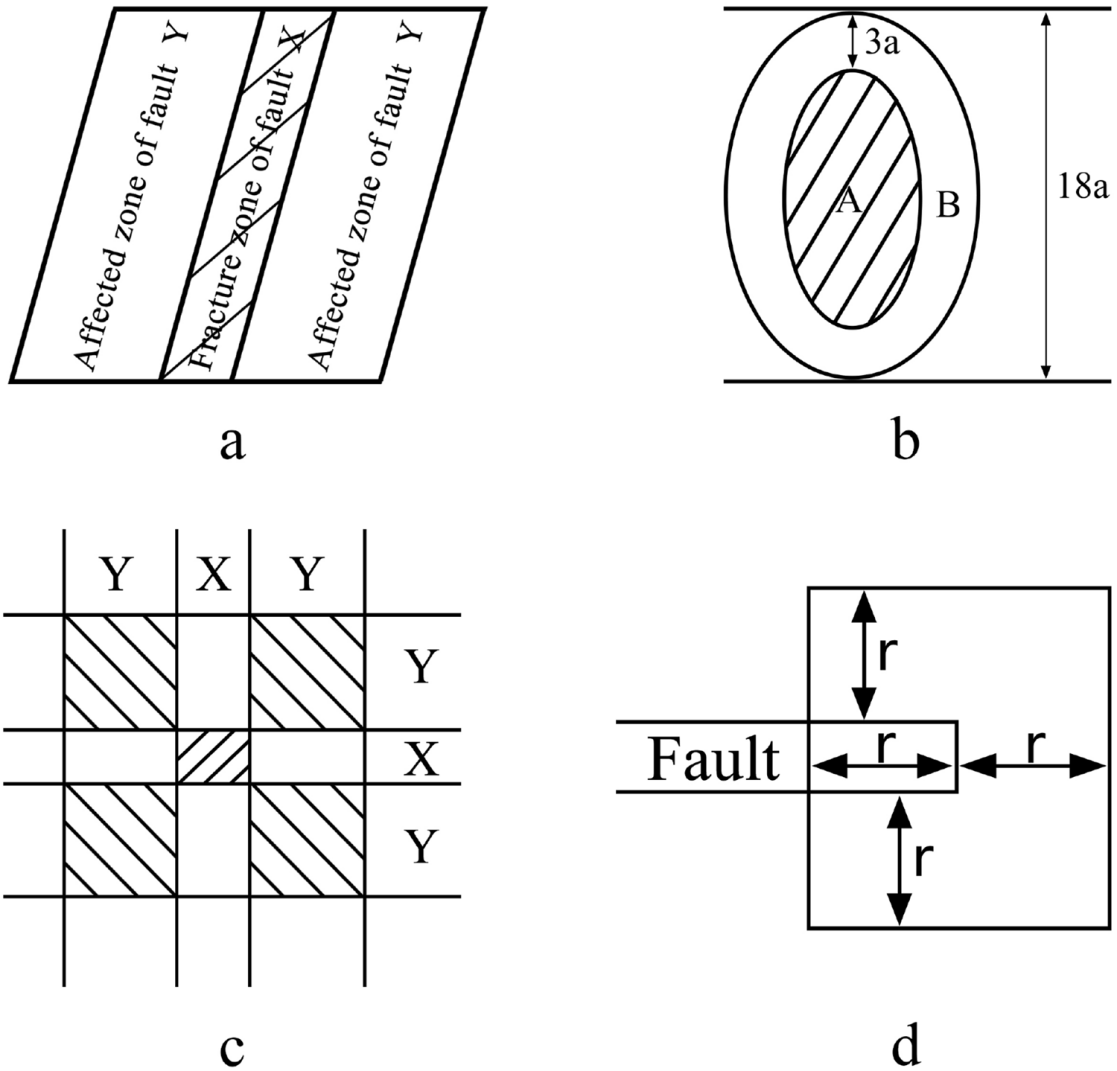
Schematic diagram of geotectonic assignment: (**a**) fault; (**b**) collapse column; (**c**) fault intersection; (**d**) fault endpoint.

Actual measurements indicate that the width of a reverse fault’s affected zone is typically greater than that of a positive fault’s affected zone, so the width of a reverse fault’s affected zone can be calculated by multiplying the actual width by 1.2. In the quantification of fault distribution, given that the fracture zone of a positive fault is more developed than an affected zone, a value of 1 is assigned to the fracture zone; consequently, 0.7 is assigned to the affected zone. Meanwhile, in reverse fault quantification, a value of 0.7 is assigned to the fracture zone, and 1 is assigned to the affected zone^19^ (Fig. 6a).

#### Distribution of collapse column

Collapse columns are primarily caused by karst collapse, and the karst strata in their distribution area are relatively well-developed. During the development of the collapse column, a channel is formed through multiple strata that provide direct communication between the coal seam and the Ordovician limestone aquifer. It allows Ordovician water to rise along the collapse column to the vicinity of the coal seam, significantly increasing the likelihood of water bursting. Additionally, the fissures formed in the surrounding rock increase the likelihood of water bursting. Based on engineering experience, the extent of the affected zone is roughly equivalent to one-sixth of the collapse column’s long-axis radius^24^. Therefore, area A is assigned the value 1 in Fig. 6b, while area B is assigned the value 0.8^19^.

#### Distribution of endpoints and intersection of fault

The faults form intersections in space, and the intersections are high-stress zones. At the end of a fault, rock fractures develop, stresses become more concentrated, and hydraulic conductivity is high when mining is susceptible to water bursting. Using the fracture zone and affected zone mentioned previously, all fault intersections and endpoints in the study area were measured and assigned a value. Figure 6c assigns a value of 2 to the region where X crosses X, a value of 1.7 to the region where X crosses Y, and a value of 1.4 to the region where Y crosses Y. The area depicted in Fig. 6d is assigned a value of 1.7^19^.

#### Fault-scale index

The fault scale index is the product of the length of the fault and the drop per unit area^25^. The value’s magnitude indicates the level of fault development in the area. The greater the value, the higher the likelihood of water bursting in the area (Eq. 6).

6 $$\mathrm{FSI}=\frac{\sum_{\mathrm{i}}^{\mathrm{n}}{\mathrm{L}}_{\mathrm{i}}{\mathrm{H}}_{\mathrm{i}}}{\mathrm{s}}$$

${\mathrm{H}}_{\mathrm{i}}$ is the fall of the fault (m); ${\mathrm{L}}_{\mathrm{i}}$ is the strike length of the fault within the unit area (m); n is the number of faults falling in the unit; and S is the unit area (300 × 300 m^2^).

## Methods

### Analytic hierarchy process (AHP)

**Figure 7:**
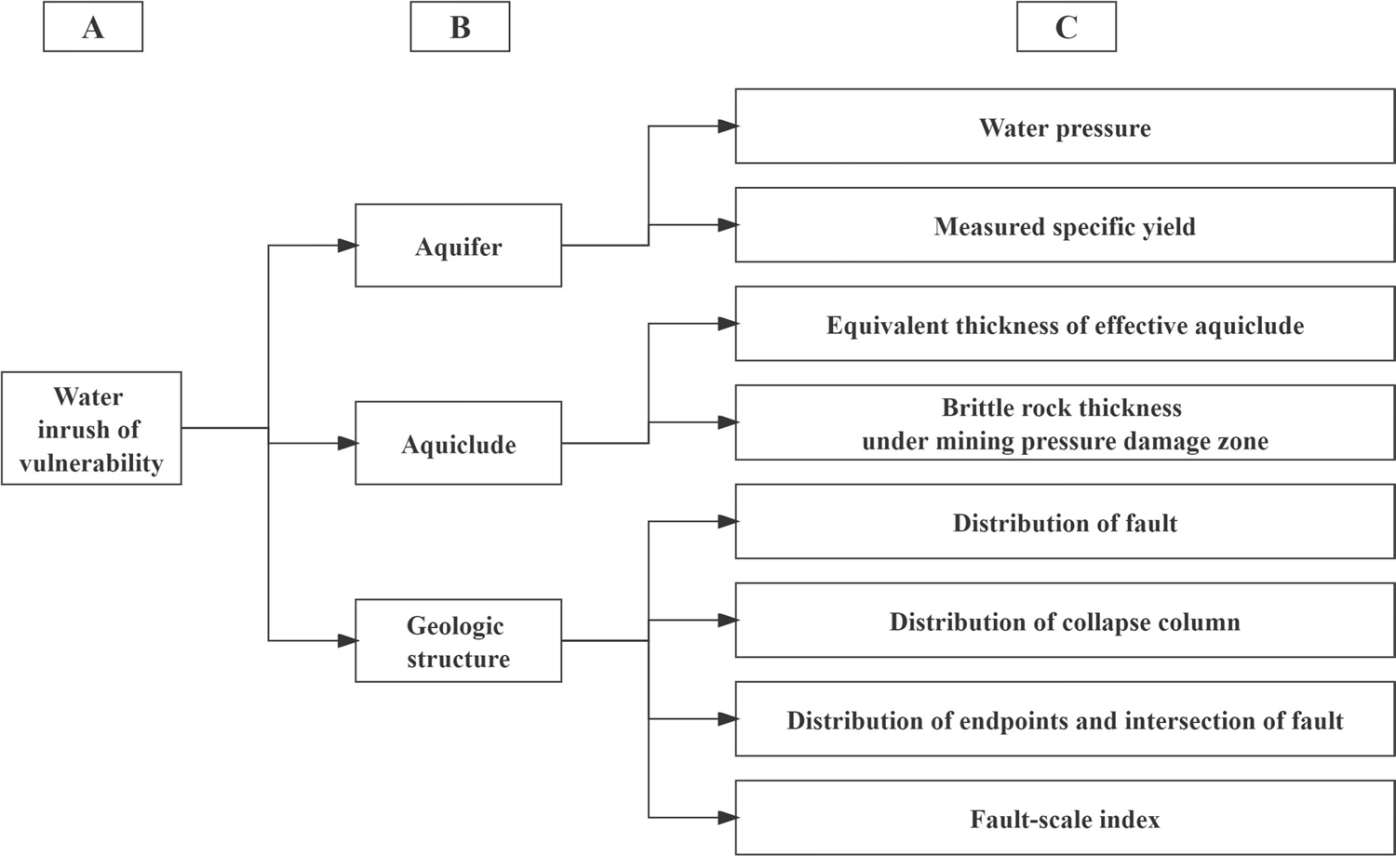
The AHP model for this study.

AHP is a popular weighting method that stratifies a complex multi-objective decision-making problem. It invites experts to score the influencing factors at each level according to their relative importance and then employs mathematical analysis to derive the weights for each influencing factor. Experts in coal mine water damage control and technicians from the Gequan mine were invited to score this study, as they have sufficient knowledge of the actual production situation to provide accurate ratings. By analysing the actual situation in the study area, the preceding section’s list of influencing factors was chosen as the weighting model’s evaluation indicators (Fig. 7).

Using the inverse scale proposed by Saaty, the final weight value of each influencing factor is determined by constructing a judgement matrix, comparing each of the two factors affecting sudden water, ranking the relative importance of each factor, numerically ranking the values representing their importance from 1 to 9, and then applying the inverse scale^15,26^.

### Data normalization

All data must be normalised to eliminate differences between influencing factors and make the data comparable across dimensions^19^. Equation (5) is the maximum method employed when a factor contributes to water bursting. Equation (6) is the minimum method for calculating when the influencing factor inhibits water bursting.

5 $${\mathrm{Y}}_{\mathrm{i}}=\frac{{\mathrm{y}}_{\mathrm{i}}-{\mathrm{y}}_{\mathrm{min}}}{{\mathrm{y}}_{\mathrm{max}}-{\mathrm{y}}_{\mathrm{min}}}$$

6 $${\mathrm{Y}}_{\mathrm{i}}=\frac{{\mathrm{y}}_{\mathrm{max}}-{\mathrm{y}}_{\mathrm{i}}}{{\mathrm{y}}_{\mathrm{max}}-{\mathrm{y}}_{\mathrm{min}}}$$

${\mathrm{Y}}_{\mathrm{i}}$ is the index value of the influencing factor nondimensionalized at point i; ${\mathrm{y}}_{\mathrm{i}}$ denotes the quantified index value of the influencing factor at point i; ${\mathrm{y}}_{\mathrm{max}}$ represents the maximum quantified index value of the influencing factor in the study area; and ${\mathrm{y}}_{\mathrm{min}}$ stands for the minimum quantified index value of the influencing factor in the study area.

**Figure 8:**
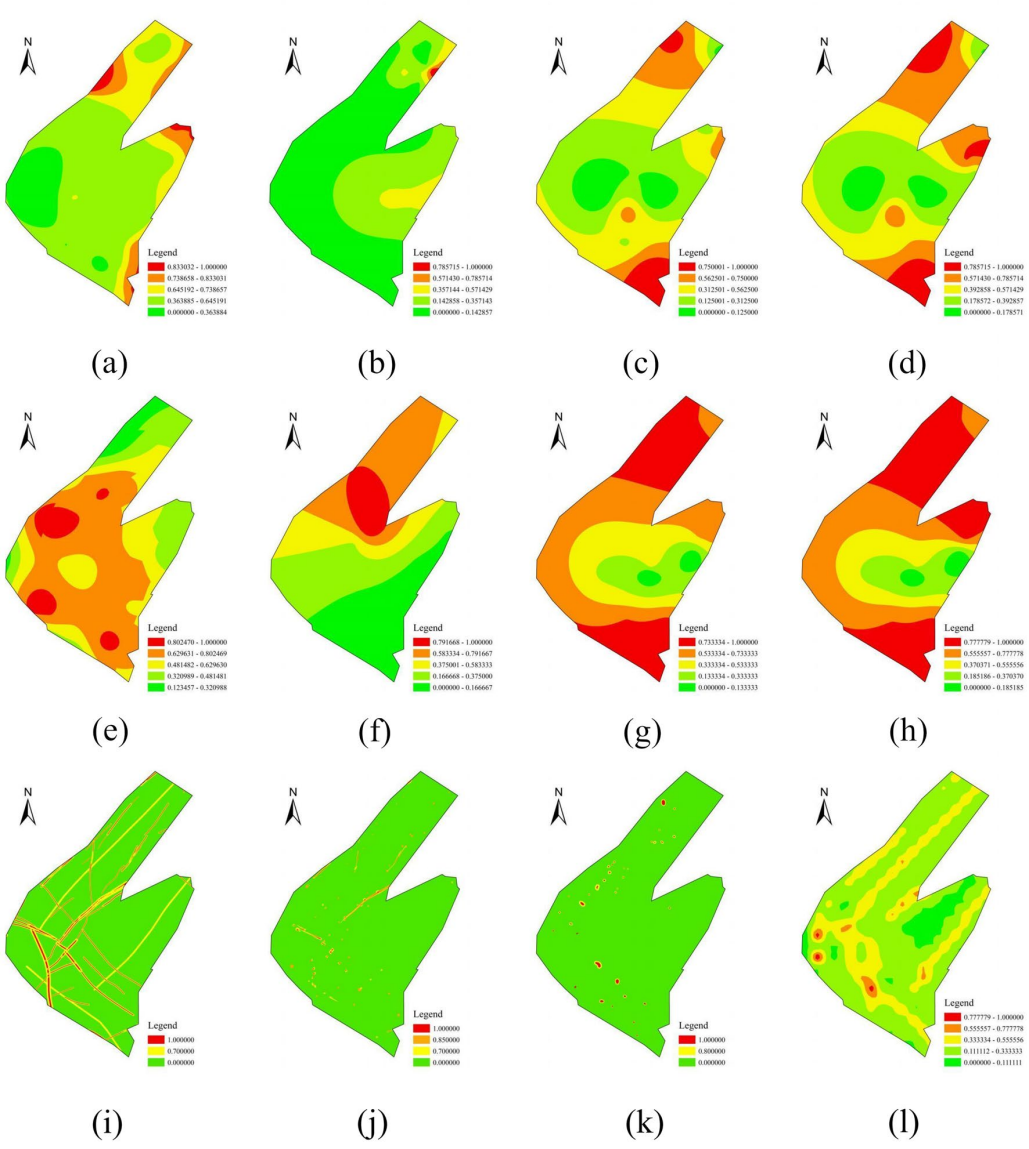
Influence factors of water bursting about the Ordovician limestone after data normalization: (**a**) water pressure; (**b**) measured specific yield; (**c**) equivalent thickness of effective aquiclude; (**d**) brittle rock thickness under mining pressure damage zone. Influence factors of water bursting about the Benxi limestone after data normalization: (**e**) water pressure; (**f**) measured specific yield; (**g**) equivalent thickness of effective aquiclude; (**h**) brittle rock thickness under mining pressure damage zone. Influence factors of water bursting about the geological structure after data normalization: (**i**) fault distribution; (**j**) collapse column distribution; (**k**) endpoint and fault intersection distributions; (**l**) fault-scale index.

Figure 8 illustrate the normalised influence factors.

### VI method

VI method is an evaluation method for water bursts that considers multiple influencing factors. GIS is used to combine normalised influencing factors and their corresponding weights^12^. Equation (7) demonstrates the calculation steps.

7 $$\mathrm{VI}=\sum_{\mathrm{i}=1}^{\mathrm{m}}{\mathrm{W}}_{\mathrm{i}}{\mathrm{f}}_{\mathrm{i}}\left(\mathrm{x},\mathrm{y}\right)$$

VI is the vulnerability index of the coal seam; ${\mathrm{W}}_{\mathrm{i}}$ denotes the weight of the influencing factors; ${\mathrm{f}}_{\mathrm{i}}\left(\mathrm{x},\mathrm{y}\right)$ refers to the single-factor influencing value function; and $\left(\mathrm{x},\mathrm{y}\right)$ represents the geographic coordinates.

## Data Availability

The datasets generated and analysed during the current study are not publicly available due keep secret but are available from the corresponding author on reasonable request.
